# Impact on sexual function after reconstructive surgery for anterior urethral stricture disease

**DOI:** 10.4103/0970-1591.65384

**Published:** 2010

**Authors:** Uday P Singh, Ruchir Maheshwari, Vikas Kumar, Aneesh Srivastava, Rakesh Kapoor

**Affiliations:** Department of Urology and Renal Transplant, Sanjay Gandhi Postgraduate Institute of Medical Sciences, Lucknow – 226 014, Uttar Pradesh, India

**Keywords:** Brief male sexual function inventory, sexual function, urethral stricture, urethroplasty

## Abstract

**Objective:**

To evaluate the effect of urethral reconstructive surgery on sexual drive, erectile function and ejaculation.

**Materials and Methods:**

The study group consisted of 150 men with a median (range) age of 40 (18–73) years who underwent 168 urethral reconstructive procedures for anterior urethral stricture disease between October 2003 and May 2009. We evaluated sexual functioning using the O’Leary Brief Male Sexual Function Inventory before and after surgery.

**Results:**

The median follow-up was 33 months (range 4–72). There were no significant changes in sexual drive and erectile function scores postoperatively for men in the 20-29, 30-39, 40-49, 50-59 and 60-69 years age groups. Overall, there was a significant improvement in ejaculatory function scores after surgery. This improvement was most robust in men in the 20-29, 30-39 and 40-49 years age group.

**Conclusion:**

Overall, anterior urethral reconstruction appears no more likely to cause postoperative sexual dysfunction. Different types of urethroplasties, surgical complexity with long stricture excision and the use of buccal graft, preputial flap/tube did not influence outcome.

## INTRODUCTION

Surgical treatment of anterior urethral strictures includes numerous options such as dilatation, direct visual internal urethrotomy (DVIU) and various reconstructive surgical techniques. Peterson and Webster[1] suggested that no one technique is appropriate for all stricture diseases and the urologist must be familiar with various surgical techniques to deal with any condition of the urethra during surgery.

These different techniques for anterior urethroplasty may involve aggressive urethral dissection extending from high in the bulbomembranous urethra to sometimes beyond the suspensory ligament. This may theoretically adversely affect the erectile function as the dissection of the urethra in the intercrural space is potentially more likely to expose erectile nerves to risk since these nerves must leave the safety of the dorsal surface of the corporeal bodies to enter the pelvis lateral to the membranous urethra behind the symphysis.

Although male urethral reconstruction has become increasingly popular and effective, to our knowledge, little is known about its potential impact on subsequent sexual performance.

To date, the impact of such surgery on potency has not been documented in Indian patients. So we retrospectively assessed the effects of anterior urethroplasty on sexual function in men who were potent preoperatively using a standardized, validated questionnaire.

## MATERIALS AND METHODS

From October 2003 to May 2009 a total of 230 patients with an average age of 40 years (range 18-73) underwent anterior urethral reconstruction performed by different reconstructive urologists at a single institute.

Patients with pelvic trauma and those not sexually active preoperatively were excluded from analysis. We used components from the validated Brief Male Sexual Function Inventory for Urology (BMFSI)[2] to evaluate the sexual function of men before and after reconstructive surgery for anterior urethral stricture disease as previously described by Erickson *et al*.[3] All data were collected after surgery in the office setting, or by telephone interviews and the preoperative data were collected on a recall basis. We did not administer questionnaires until at least three months postoperatively. We used all the questions from the BMFSI that evaluated ejaculatory function (EjF) (two questions), sexual drive (SD) (two questions) and erectile function (EF) (three questions), slightly modifying the wording of the original questions to correspond to the specific interval before and after urethral surgery (Appendix).[2] The scores for each question ranged from 0 to four (0, poor overall function; four, excellent function) within each domain. As proposed by O’Leary *et al*,[2] we summed scores within each domain for a possible total of eight points in the EjF domain, 12 in the EF domain and eight in the SD domain. To compare outcomes with normative data, study participants were stratified by age using the method of O’Leary *et al*,[2] using the ranges 20-29, 30-39, 40–49, 50–59 and 60–69 years. Men aged < 20 years and > 69 years were included in the 20-29 and 60-69 years age group respectively. We used the paired Student’s *t*-test to compare data before and after surgery, and used a multivariate analysis, using stricture length and the type of repair as variables that might affect sexual functioning after surgery. The level of statistical significance was set at *P* < 0.05. All calculations were performed with statistical software SPSS^®^ 13.0 (SPSS Inc, Chicago, IL).

## RESULTS

Of 230 men eligible for inclusion, 150 (65%) completed the BMFSI for a total of 168 reconstructive procedures. The etiology of stricture was traumatic in 22 patients, idiopathic in 98, iatrogenic in 22 and lichen sclerosis in eight. Stricture location was penile in eight patients, penobulbar in 47, and bulbar in 95. The operations included stricture excision and end-to-end anastomosis (59 of 150, 39%), urethral reconstruction with dorsal onlay of buccal mucosa (55 of 150, 37%), and penile skin flap/tube urethroplasty (36 of 150, 24%). Subjects underwent an average of 0.30 DVIU (range 0 to four) prior to undergoing urethroplasty. The mean (range) patient age at surgery was 40 (18–73) years. The mean (SD) stricture length for all repairs was 4.34 ± 2.9 cm. The end-to-end anastomotic group had significantly less stricture length as compared to the other groups.

Table 1 shows the mean BMFSI scores within each sexual function domain before and after surgery. When all age groups were combined, there were no significant differences in the mean scores within the SD and EF domains before and after surgery.

**Table 1:** The mean BMFSI scores

BMFSI domain	Score, mean (SD)		Age reference range,	*P*
	Before surgery	After surgery	Mean (SD)^†^	
SD (range 0-8)				
All ages	5.5 (1.8)	5.3 (1.9)		0.50
Age group, years (n)				
20-29 (39)	7.1 (0.5)	7.0 (0.6)		0.73
30-39 (32)	6.9 (0.8)	6.8 (1.0)		0.69
40-49 (35)	5.3 (0.8)	5.0 (0.9)	5.2 (1.5)	0.23
50-59 (29)	3.8 (0.7)	3.6 (0.9)	4.5 (1.6)	0.45
60-69 (15)	2.1 (0.9)	1.9 (0.7)	3.7 (1.7)	0.52
EF (range 0-12)				
All ages	9.1 (2.5)	8.8 (2.7)		0.39
Age group, years (n)				
20-29 (39)	10.9 (1.1)	10.8 (1.1)		0.70
30-39 (32)	10.6 (1.2)	10.4 (1.4)		0.46
40-49 (35)	9.5 (1.5)	9.2 (1.8)	9.8 (2.5)	0.47
50-59 (29)	7.3 (1.6)	7.0 (1.5)	8.8 (3.0)	0.37
60-69 (15)	3.8 (0.7)	3.4 (0.9)	6.5 (3.8)	0.22
EjF (range 0-8)				
All ages	4.7 (1.2)	6.3 (1.6)		<0.001*
Agegroup, years (n)				
20-29 (39)	4.8 (1.0)	7.4 (0.6)		<0.001*
30-39 (32)	5.0 (1.1)	7.2 (0.7)		<0.001*
40-49 (35)	5.1 (1.0)	6.8 (0.8)	7.4 (1.4)	<0.001*
50-59 (29)	4.8 (0.9)	5.1 (0.8)	7.0 (1.7)	0.22
60-69 (15)	2.6 (0.8)	2.7 (0.8)	5.9 (2.7)	0.67

* statistically significant; †from O’Leary *et al*.[4]

**Appendix:** 

The BMFSI from O’Leary *et al*.[2]	
**Sexual Drive**	
Let’s define sexual drive as a feeling that may include wanting to have a sexual experience (masturbation or intercourse), thinking about having sex, or feeling frustrated due to lack of sex	
1. During the past 30 days/30 days before surgery, on how many days have you felt sexual drive?	
No days	0/0
Only a few days	1/1
Some days	2/2
Most days	3/3
Almost every day	4/4
2. During the past 30 days/30 days before surgery, how would you rate your level of sexual drive?	
None at all	0/0
Low	1/1
Medium	2/2
Medium high	3/3
High	4/4
**Erections**	
3. Over the past 30 days/30 days before surgery, how often have you had partial or full sexual erections when you were sexually stimulated in any way?	
Not at all	0/0
A few times	1/1
Fairly often	2/2
Usually	3/3
Always	4/4
4. Over the past 30 days/30 days before surgery, when you had erections, how often were they firm enough to have sexual intercourse?	
Not at all	0/0
A few times	1/1
Fairly often	2/2
Usually	3/3
Always	4/4
5. How much difficulty did you have getting an erection during the past 30 days/ 30 days before surgery?	
Did not get erections at all	0/0
A lot of difficulty	1/1
Some difficulty	2/2
Little difficulty	3/3
No difficulty	4/4
**Ejaculation**	
6. In the past 30 days/30 days before surgery, how much difficulty have you had ejaculating when you have been sexually stimulated?	
Have had no sexual stimulation in the past month	0/0
A lot of difficulty	1/1
Some difficulty	2/2
Little difficulty	3/3
No difficulty	4/4
7. In the past 30 days/30 days before surgery, how much did you consider the amount of semen you ejaculate to be a problem for you?	
Did not climax	0/0
Big problem	1/1
Medium Problem	2/2
Small problem	3/3
No problem	4/4

When we stratified SD and EF score by age, men aged 50-59 and 60-69 years, were having low level of SD and EF domain score preoperatively as well as postoperatively as compared to normative levels for community-based men aged 50-59 and 60-69 years established by O’Leary *et al*.[4][Table 1].

In the EjF domain, there was a significant increase in the overall EjF score after surgery for the operative group as a whole (*P* < 0.001). When we stratified scores by patient age, men of 20-29, 30, 39 and 40-49 years age group, had a significantly higher postoperative EjF score as compared to preoperative score. But there was no significant change in EjF score for men of the 50-59 and 60-69 years age group, postoperatively, and these men also had low EjF score as compared to normative levels for community-based men of 50-59 and 60-69 years age group, established by O’Leary *et al*.[4]

Multivariate analysis showed that neither the type of stricture repair nor the length of stricture significantly altered overall EF, SD or EjF.

## DISCUSSION

We found that different types of anterior urethroplasties whether associated with short or long strictures did not influence postoperative outcome with respect to EF.

When we simply compared BMFSI scores before and after surgery, there were no significant changes in SD and EF scores for men of 20-29, 30-39, 40-49, 50–59 and 60-69 years age groups [Table 1]. However, SD and EF scores before and after surgery for men aged ≥40 years were lower than the normative levels for community-based men aged ≥40 years as established by O’Leary *et al*. [Table 1].

The normative data from O’Leary did not include any men aged ≤ 40 years. In our study, the younger age group (< 40 years) showed good erectile function and sexual drive before as well as after surgery.

Overall, there was a significant improvement in EjF scores after surgery. This improvement was most robust in men of 20-29, 30-39, and 40-49 years age groups. The mean (SD) EjF domain score for men of the 20-29, 30-39, and 40-49 years age groups before surgery and after surgery was 4.8 (1.0), 5.0 (1.1), 5.1 (1.0) and 7.4 (0.6), 7.2 (0.7), 6.8 (0.8) respectively, similar to the age reference range of 7.4 (1.4). In men aged 50-59 and 60–69 years the EjF scores were lower as compared to normative community data and did not significantly change after surgery.

Using the O’Leary BMSFI, Erickson *et al*.[3] found that only men 50 to 59 years old, compared to younger men, had decreased EF after surgery. We had similar findings, although the majority of the older men in our study were having low level of sexual drive and erectile function before surgery. But improvement in mean EjF scores in young patients was similar. As proposed by Erickson *et al*. the improvement in mean EjF score may be due to the relief of the urethral obstruction, and resection of the acontractile, scarred segment of the urethra/spongiosum which improves the rhythmic expulsion mechanism by re-establishing the continuity of the musculature. They also proposed that objective data, such as semen volume measurement, might be a better way to evaluate ejaculatory function.

There appears to be little anatomical basis for severe erectile dysfunction after anterior urethral reconstruction. According to Lue *et al*.[5] some cavernous fibers pass through the tunica albuginea to supply the corpus spongiosum but most cavernous fibers remain approximately three mm outside of the corpus spongiosum at the one and 11 o’clock positions. Our finding that patients who underwent anterior urethroplasty reported the least decrement in erectile satisfaction corroborates the conclusion of Lue *et al*.[5] that the corpus spongiosum conducts only a minority of the overall erectile neural mechanism.

In our study, 79% patients reported equal or improved EF score postoperatively and the remaining 21% patients although reported lower absolute value of EF scores, the decrease was not statistically significant for different age groups as well as for this cohort as a whole. Similar to the findings of our study, a retrospective mailed questionnaire study of 152 men by Coursey *et al*.[6] showed that anterior urethroplasty was no more likely to cause long-term postoperative sexual dysfunction than circumcision.

Nelson *et al*.[7] did buccal mucosal graft urethroplasty for previously failed hypospadias repair and found that long-term sexual function and satisfaction was excellent in a small group of men. Although our patient population was older (median age 40 vs. 17.5 years), in our study the men of the younger age group also had excellent postoperative EF. The data from the two studies together suggest that anterior urethroplasty, whether involving the penile or bulbar urethra, has an insignificant effect on EF, even when the diseased portion of the urethra is extensive and the use of a graft is required.

Jennifer *et al*.[8] did a prospective study on a cohort of 25 patients and found that surgical complexity with long stricture excision and the use of a buccal graft did not influence postoperative outcome with respect to EF.

The limitations of the present study include the nature of the preoperative sexual function assessment (retrospective) and cross-sectional design. We also acknowledge that, because preoperative data were gathered retrospectively, these data are subject to recall bias. All efforts were made during data collection to ensure that accurate preoperative assessments were being made, but nonetheless, the preoperative data must be interpreted carefully. Despite these weaknesses, we strongly feel that the information collected from patient-reported data rather than physician-reported impressions provides more insight than is currently available into the specific type of sexual dysfunction after reconstructive surgery.

## CONCLUSION

Using the BMFSI, we showed that urethral reconstructive surgery does not significantly affect EF or SD but it improves EjF significantly in the younger age group. Surgical complexity with long stricture excision and the use of a buccal graft did not influence outcome. However, in our study older men had lower level of sexual and erectile function before surgery as compared to the normative community reference range for the same age group as proposed by O`Leary. Fully understanding the changes in sexual function after reconstructive surgery for anterior urethral stricture disease requires prospective studies.
